# Functional characterization of BbEaf6 in *Beauveria bassiana*: Implications for fungal virulence and stress response

**DOI:** 10.1080/21505594.2024.2387172

**Published:** 2024-07-31

**Authors:** Qing Cai, Juan-Juan Wang, Jia-Tao Xie, Dao-Hong Jiang

**Affiliations:** a State Key Laboratory of Agricultural Microbiology, College of Plant Science and Technology, Huazhong Agricultural University, Wuhan, Hubei, China; b School of Biological Science and Biotechnology, University of Jinan, Jinan, Shandong, China

**Keywords:** Entomopathogenic fungus, histone acetyltransferase, gene transcription, asexual development, virulence, stress response

## Abstract

The Eaf6 protein, a conserved component of the NuA4 and NuA3 complexes in yeast and MOZ/MORF complexes in humans, plays crucial roles in transcriptional activation, gene regulation, and cell cycle control. Despite its significance in other organisms, the functional role of Eaf6 in entomopathogenic fungi (EPF) remained unexplored. Here, we investigate the function of BbEaf6, the Eaf6 homolog in the entomopathogenic fungus *Beauveria bassiana*. We demonstrate that BbEaf6 is predominantly localized in nuclei, similar to its counterpart in other fungi. Deletion of *BbEaf6* resulted in delayed conidiation, reduced conidial yield, and altered conidial properties. Transcriptomic analysis revealed dysregulation of the genes involved in asexual development and cell cycle progression in the Δ*BbEaf6* mutant. Furthermore, the Δ*BbEaf6* mutant exhibited decreased tolerance to various stresses, including ionic stress, cell wall perturbation, and DNA damage stress. Notably, the Δ*BbEaf6* mutant displayed attenuated virulence in insect bioassays, accompanied by dysregulation of genes associated with cuticle penetration and haemocoel infection. Overall, our study elucidates the multifaceted role of BbEaf6 in stress response, development, and virulence in *B. bassiana*, providing valuable insights into the molecular mechanisms governing fungal pathogenesis and potential targets for pest management strategies.

## Introduction

Histone acetylation stands as one of the most prevalent histone post-translational modifications (PTMs), which is mediated by histone acetyltransferases (HATs) [1]. Typically, HATs are capable of adding acetyl groups to specific histone residues, which results in chromatin decondensation and gene activation [2,3]. The NuA4 complex, a highly conserved histone acetyltransferase (HAT) complex spanning from yeast to humans, demonstrates a preference for acetylating histones H4 and H2A [4,5]. The NuA4 complex plays a pivotal role in various genomic processes, including ensuring chromosome stability, facilitating gene transcription, participating in DNA damage repair, and promoting cell cycle progression [6–8]. Beyond its established role in modifying histones, the NuA4 complex showcases remarkable versatility by acetylating over 250 non-histone substrates, allowing NuA4 to exert critical control over cellular metabolism, autophagy, and homoeostasis [9,10]. Another similar histone acetyltransferase complex, called the NuA3 HAT complex, mainly promotes acetylation of histone H3 lysine 14 (H3K14) and mediates transcription of a subset of downstream genes in yeast [11].

Eaf6 homologs are found across various organisms, serving as integral components of NuA4 and NuA3 complexes in yeast, as well as MOZ and MORF complexes in humans [12–14]. Notably, the deletion or mutation of yeast Eaf6 does not lead to lethal phenotypes, suggesting that this NuA4 subunit is not indispensable for yeast [15]. However, in the NuA3 complex, yeast Eaf6 contributes to transcriptional activation and cell cycle regulation by facilitating H3 acetylation [11,16]. In humans, Eaf6 forms trimeric complexes with ING5 and BRPF, playing crucial roles in cell proliferation, disease development, and interactions with MOZ and MORF complexes [14,17]. In *Arabidopsis thaliana*, Eaf6 functions as a characteristic subunit of the NuA4 complex and is essential for chlorophyll biosynthesis and photosynthesis [18]. Moreover, the deletion of Eaf6 in *Oryza sativa* can lead to a hybrid breakdown in intersubspecific rice crosses [19]. Additionally, Eaf6 homolog has been identified in the fungal pathogen *Fusarium graminearum*, which causes Fusarium head blight (FHB) or scab on wheat [20]. In this pathogen, FgEaf6 has been shown to positively regulate virulence, asexual/sexual reproduction, and conidial septation, the deletion of *FgEaf6* significantly impairs the sporulation process and pathogenicity on wheat [20].

Despite its significance in other organisms, however, the functions of Eaf6 homologs in other fungi, particularly entomopathogenic fungi, remain largely unexplored. One such example is the insect pathogenic fungus, *Beauveria bassiana*, widely recognized as a fungal biological pest control agent globally [21]. In *B. bassiana*, the efficacy of biological control hinges on critical aspects such as conidial production, tolerance to various stresses, and pathogenicity against insect hosts [22,23]. With the completion of genome sequencing of *B. bassiana*, an expanding number of related activators have been characterized, including asexual developmental activators, antioxidant enzymes, cuticle-degrading enzymes, etc. [24,25]. Moreover, in this fungus, we also investigated the global acetylation network previously, which not only emphasized the importance of lysine acetylation, but also provided more genetic targets to explore [26]. So far, in *B. bassiana*, five histone acetyltransferases have been characterized, comprising three GNAT superfamily members (Gcn5, Spt10, Elp3), one MYST superfamily member (Mst2) and one P300/CBP family member (Rtt109), showcasing diverse impacts on developmental and virulence pathways within the fungus [27–31]. Here, we elucidate that the *B. bassiana* NuA4 subunit Eaf6 is vital for fungal sporulation, stress responses, and pathogenicity. The deletion of *BbEaf6* resulted in decreased conidiation, conidial resistance to multi-stress and attenuated virulence upon insect hosts. Correspondingly, comparative transcriptomic analyses also revealed a large array of related genes, including asexual developmental activators, cuticle-degrading enzymes and many different kinds of cellular transporters.

## Materials and methods

### Blast search and identification of Eaf6 homolog in B. bassiana

The BLAST research using the amino acid sequence of *Saccharomyces cerevisiae* Eaf6 protein (ScEaf6, NP_012615) was conducted to identify homologs in the genomic database of *B. bassiana* ARSEF 2860 (NCBI accession: NZ_ADAH00000000) [24], as well as in other representative fungi, including fungal pathogens of plants, insects, and humans (http://blast.ncbi.nlm.nih.gov/). Subsequently, the identified sequences were aligned using the SMART program (http://smart.embl-heidelberg.de) to facilitate structural comparison. Phylogenetic analysis was then conducted using MEGA7 software (http://www.megasoftware.net) to assess evolutionary relationships. Additionally, the molecular weight and isoelectric point (pI) of BbEaf6 were predicted using the ExPASy-Compute pI/Mw tool (https://web.expasy.org/compute_pi/).

### Subcellular localization of BbEaf6 protein

The subcellular localization of the Eaf6 homolog in *B. bassiana* (BbEaf6) was investigated using a fusion protein approach. The coding region of BbEaf6 was amplified from wild-type cDNA using specific primers (Eaf6-LC-F/R, see Table S1) and fused to the green fluorescent protein (GFP) at the C-terminus of BbEaf6. This fusion gene, BbEaf6:GFP, was then cloned into plasmid pAN52-bar, which confers resistance to phosphinothricin. The BbEaf6:GFP fusion construct was integrated into the genome of the wild type *B. bassiana* strain via blastospore transformation, following a protocol described elsewhere [32]. Transgenic colonies expressing the fusion protein were identified by their resistance to phosphinothricin (200 μg/ml). The localization of the fusion protein was visualized using confocal microscopy. For visualization, conidia were cultured in SDB (4% glucose, 1% peptone, and 1% yeast extract) at 25°C for 3 days with aeration at 150 rpm. Hyphal cells were then collected and counter-stained with DAPI (nuclei-specific dye, Sigma) for 30 minutes at room temperature before microscopic examination.

### Construction of B. bassiana Eaf6 deleted and complemented strains

To generate a targeted gene deletion strain of *BbEaf6* (Δ*BbEaf6*) in *B. bassiana*, homologous recombination was employed using 5’and 3’ coding fragments (1509 and 1442 bp, respectively) flanking the *bar* selection marker followed a strategy previously described [27]. Subsequently, a complemented strain (Δ*BbEaf6:BbEaf6*) was constructed through ectopic integration of the full-length *BbEaf6* sequence, along with approximately 1500 bp of the 5’-flanking (promoter) region (totalling 3528 bp), cloned into a vector containing the *sur* sulfonylurea resistance marker. DNA fragments required for these manipulations were amplified from *B. bassiana* genomic DNA using paired primers (Table S1) and La*Taq* DNA polymerase. Putative Δ*BbEaf6* mutant colonies were screened either by bar-mediated resistance to phosphinothricin (200 μg/ml) or by sur resistance to chlorimuron ethyl (10 μg/ml) for the Δ*BbEaf6:BbEaf6* strain. Corrected integration events were verified through PCR and quantitative PCR (qPCR), employing the primers and procedures outlined in Table S1.

### Investigation of fungal tolerance to multi-stresses

To evaluate responses to various stressors, 1 μl aliquots of 1 × 10^6^ conidia/ml suspensions from each strain were spotted onto different agar media plates. The base medium used was Czapek Dox Agar (CDA, comprising 3% sucrose, 0.3% NaNO_3_, 0.1% K_2_HPO_4_, 0.05% KCl, 0.05% MgSO_4_, and 0.001% FeSO_4_, supplemented with 1.5% agar). Additionally, 11 CDA-derived media were prepared, each supplemented with different stress-inducing agents: (i) Osmotic Stresses: 0.4 M NaCl or KCl; (ii) Ionic Stresses: 2 mM CuCl_2_, 4 mM MnCl_2_ or FeCl_2_; (iii) Oxidative Stresses: H_2_O_2_ (2 mM) or menadione (0.02 mM); (iv) SDS (100 μg ml^−1^) or Congo red (10 μg ml^−1^) for cell wall perturbing stresses; (v) Cell Wall Perturbing Stresses: hydroxyurea (HU, 10 mM) or methyl methanesulfonate (MMS, 0.05%). After incubation for 8 days at 25°C, the diameter of each colony was measured. The relative growth inhibition of each strain by each chemical was calculated using the formula: (*S*_c_–*S*_t_)/*S*_c_ ×100, where *S*_*c*_ represents the area of the control colony and *S*_*t*_ represents the area of the stressed colony.

### Examination of fungal conidiation and conidial activity

To assess conidiation capacity, aliquots of 100 μl from a 1 × 10^7^ conidia/ml suspension were spread on SDAY plates (9 cm diameter) and incubated for 9 d at 25°C under a 12:12-hour light/dark cycle. Beginning from day 4 of incubation, three plugs (5 mm diameter) were extracted daily from each plate, and the conidia from each plug were harvested into 1 ml of 0.02% Tween 80 using ultrasonication. The conidial suspensions were then counted using a haemocytometer, and the conidial concentrations were converted to the number of conidia per square centimetre (conidia/cm^2^). In addition, the sporophore structures on SDAY plates from days 3 to 6 post-inoculation were morphologically examined under microscopy. The size and density (complexity) of 7-day-old aerial conidia (2 × 10^4^ conidia per sample) were quantified according to the forward scatter (FSc) and side scatter (SSc) readings in the flow cytometry. Conidial viability was assessed by determining the time for 50% germination of conidia (GT_50_) at 25°C.

### Insect bioassays to test the fungal virulence

The insect bioassays were conducted using *Galleria mellonella* larvae with two distinct methods of infection. (i) Topical Bioassays: Approximately 30 larvae were immersed for 10 seconds in 30 ml of a suspension containing 1 × 10^7^ conidia/ml for cuticular infection. (ii) Intra-hemocoel Assays: For each larva, 5 μl of a suspension containing 1 × 10^5^ conidia/ml was injected directly into the hemocoel. Following immersion or injection, the larvae were transferred to individual containers and maintained at 25°C for a duration of 10 days. Survival of the larvae was monitored at regular intervals, either every 12 or 24 hours. The bioassay experiments were repeated three times to ensure reliability and consistency. Probit analysis was employed to estimate the mean lethal time (LT_50_) required to kill 50% of the hosts based on time-mortality trends. Additionally, after 4 days post-death, fungal hyphae growing on the surfaces of dead larvae were observed and examined.

To investigate the fungal growth and proliferation dynamics, biomass levels (comprising blastospores and hyphae) and blastospore concentrations were quantified from 3-day-old submerged cultures initiated with 50 ml aliquots of a 10^6^ conidia/ml suspension in two different media: CZB (standard medium) and TPB (an amended CZB mimic to insect haemolymph, containing 3% trehalose as the sole carbon source and 0.5% peptone as the sole nitrogen source). The obtained biomass levels and blastospore concentrations were utilized to estimate the dimorphic transition rate (N blastospores/mg), which serves as a reference to the speed of fungal proliferation within the host haemocoel.

### Examination of cell cycle and hyphal septation pattern

Hyphae were harvested from cultures inoculated with 50 ml of 1 × 10^6^ conidia/ml suspension in Sabouraud Dextrose Broth (SDB) and shaken at 150 rpm for 3 days at 25°C. Subsequently, the hyphae were stained with calcofluor white (Sigma) for 15 minutes and examined for septation pattern and cell morphology under a fluorescent microscope. The mean unicellular cell length and width were examined from approximately 30 stained multicellular hyphal cells of each strain using ImageJ software. To facilitate the production of unicellular blastospores for cell cycle analysis, aliquots of 50 ml of a 1 × 10^6^ conidia/ml suspension were inoculated into Nutrient Limited Broth (NLB) containing 4% glucose, 0.4% NH_4_NO_3_, 0.3% KH_2_PO_4_ and 0.3% MgSO_4_. These cultures were then incubated by shaking at 150 rpm for 3 days at 25°C. Blastospores collected from NLB cultures were used to determine the G0/G1, G2/M, and S phases of the cell cycle based on the respective readings of unduplicated (1C), duplicated (2C), and intermediate DNA concentrations obtained from flow cytometry analysis (FACS) of three samples per strain. Additionally, blastospore size and density were assessed using the FSc and SSc readings from the flow cytometry, respectively.

All phenotypic data above were subjected to a one-way ANOVA analysis. Post hoc comparisons were performed using Tukey’s honestly significant difference (HSD, *p* < 0.05) test to determine significant differences between experimental groups.

### Genome wide transcriptome analysis

To investigate the potential gene targets and downstream process regulated by BbEaf6, RNA-Seq analyses were conducted using three replicates of 4-day cultures of the Δ*BbEaf6* strain and wild-type strain (WT) grown on cellophane-overlaid SDAY plates. The cells were harvested using sterilized spoons, and frozen cells were sent to Personal Biotechnology Co., Ltd. (Shanghai, China) for the construction and analysis of transcriptomes. Total RNA extraction was performed using RNA Trizol (Sigma), and mRNAs were isolated from total RNAs using magnetic oligo(dT) beads. The isolated mRNAs were fragmented into segments using the ionic disruption method, and the mRNA fragments served as templates for synthesizing the first-strand cDNAs using random hexamer primers. Subsequently, second-strand cDNAs were synthesized using a cDNA Synthesis Kit (Sigma) with the first-strand cDNAs as templates. Each double-stranded cDNA was purified and end-repaired, with single adenines added to the ends of the cDNA molecules. Finally, a cDNA library was constructed by adding appropriate adaptors to the cDNA. The samples were sequenced on an Illumina HiSeq platform. All raw reads obtained from sequencing the cDNA samples were filtered to generate clean tags, which were then mapped to the genome of *B. bassiana* [32] with significant levels of log2(Δ*BbEaf6*/WT ratio) < −1 (down-regulated) or >1 (up-regulated), and false discovery rate (FDR) <0.01. All identified Differentially Expressed Genes (DEGs) were functionally annotated with known or putative gene information from non-redundant NCBI protein databases and subjected to Funcat category classification (https://elbe.hki-jena.de/fungifun/). Additionally, Gene Ontology (GO) enrichment analyses (https://geneontology.org) were performed to enrich the DEGs into various GO categories at a significant level of *p* < 0.05.

## Results

### Characterization of BbEaf6, and construction of deletion and complemented strains

Based on BLAST research with yeast Eaf6 (NP_012615) as a query, the Eaf6 homolog was identified in *B. bassiana* ARSEF 2860 (XP_008601226). In the *B. bassiana* genomic dataset, BbEaf6 is comprised of a nucleotide sequence of 706 bp containing two introns. The open reading frame (ORF) of BbEaf6 can be translated into a protein of 182 amino acids, with a predicted molecular mass as 19.4 kDa (isoelectric point: 10.03). In terms of the conserved domains, BbEaf6 has only one called NuA4 superfamily domain (residues 28–102), which typically presented in NuA4 subunit superfamily members. Through protein alignment analysis, this conserved domain was also founded in Eaf6 homologs in *F. graminearum*, *Candida albicans*, and *S. cerevisiae* (Figure S1(a)). Phylogenetic relationship of BbEaf6 with other homologs in representative fungi was also conducted. As showed in the phylogenetic tree (Figure S1(b)), BbEaf6 shared high sequence identities (~60–83%) with homologs found in filamentous fungi, while displaying relatively lower identities (~25–35%) with yeasts, indicating specific roles for Eaf6 homologs in filamentous fungi.

**Figure 1.**
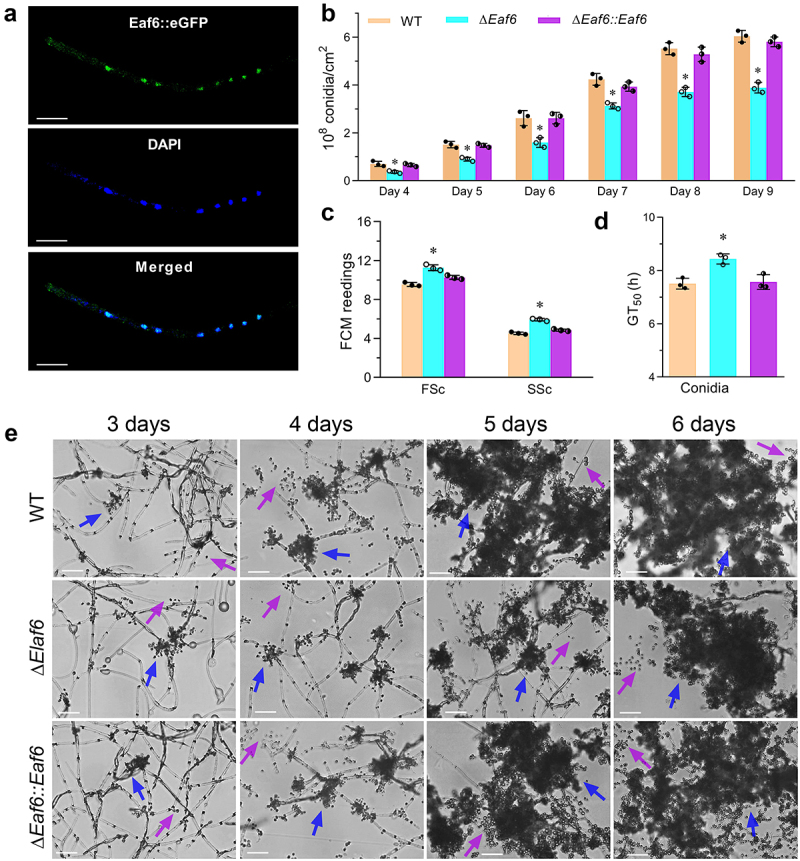
Subcellular localization of BbEaf6 and its role on conidiation and conidial properties in *B. bassiana*.

To assess the subcellular localization of BbEaf6, the full coding region of BbEaf6 protein was amplified and fused to the green fluorescent protein (GFP) at the C-terminus of BbEaf6, then a transgenic strain expressing a BbEaf6:GFP fusion protein was generated. After 3-day growth in SDB, the hyphal cells of the transgenic strain were collected from 72-hour Sabouraud dextrose broth (SDB) cultures and stained with the nuclei-specific dye DAPI. As showed in the fluorescence images in Figure 1(a), the green fluorescence of BbEaf6 was mainly observed in the nuclei of hyphal cells, which overlap well with the blue fluorescence of the nuclei-specific dye DAPI, indicating predominant nuclear localization of BbEaf6 (Figure 1(a)).

(a) Laser scanning confocal microscopy (LSCM) images (scale bars: 10 µm) depicting the subcellular localization of GFP-tagged Eaf6 fusion protein expressed in 3-day-old wild type (WT) cells. (b) Quantification of conidial yields during 4 to 9-day cultivation on SDAY plates. (c) Flow cytometry analysis for cell size (FSc) and density (SSc) of aerial conidia collected from 7-day-old SDAY cultures. (d) Assessment of conidial viability indicated by the time required for 50% germination (GT_50_). (e) Microscopic visualization of growing of the sporophores (pointed by blue arrows) and conidia (pointed by purple arrows) on SDAY plates during 3–6-day cultivation (scale bars = 20 µm).

All experiments were performed in triplicate (Tukey’s honestly significant difference test, *p* < 0.05).

In order to further investigate the function of BbEaf6, the *BbEaf6* single-gene deletion (Δ*BbEaf6*) and complemented (Δ*BbEaf6:BbEaf6*) strains were constructed and verified via PCR and quantitative PCR analyses (Figure S2, primers listed in Table S1).

### Impact of Eaf6 deletion on sporulation and conidial yield

To elucidate the role of BbEaf6 in sporulation processes, conidial suspensions of all strains, including wild type, deletion mutant (Δ*BbEaf6*), and complemented strain (Δ*BbEaf6:BbEaf6*), were spread on SDAY plates and incubated for 9 days. As showed in Figure 1(b), the Δ*BbEaf6* strain exhibited significantly decreased conidial production, with reduced conidial yields by ~ 39–48% after 4–6 days post-inoculation and ~ 26–36% after 7–9 days post-inoculation. Microscopic examination also revealed significantly fewer sporophores (pointed by blue arrows) and conidia (pointed by purple arrows) in the Δ*BbEaf6* mutant compared to control strains (Figure 1(e)).

In terms of the conidial properties, as indicated in the results of FACS (Fluorescence activated Cell Sorting) analyses (Figure 1(c)), compared to wild type, the conidial size was significantly affected in the Δ*BbEaf6* mutant, with an ~18% increase in conidial size and a ~ 31% increase in conidial density. However, the conidial germination was significantly impaired, and the time required for 50% of the conidia to germinate (GT_50_) was prolonged by ~12% for Δ*BbEaf6* cells (Figure 1(d)). All of these results indicated the important role of BbEaf6 in sporulation and conidial germination.

### Contribution of BbEaf6 in fungal tolerance to multi-stress

**Figure 2.**
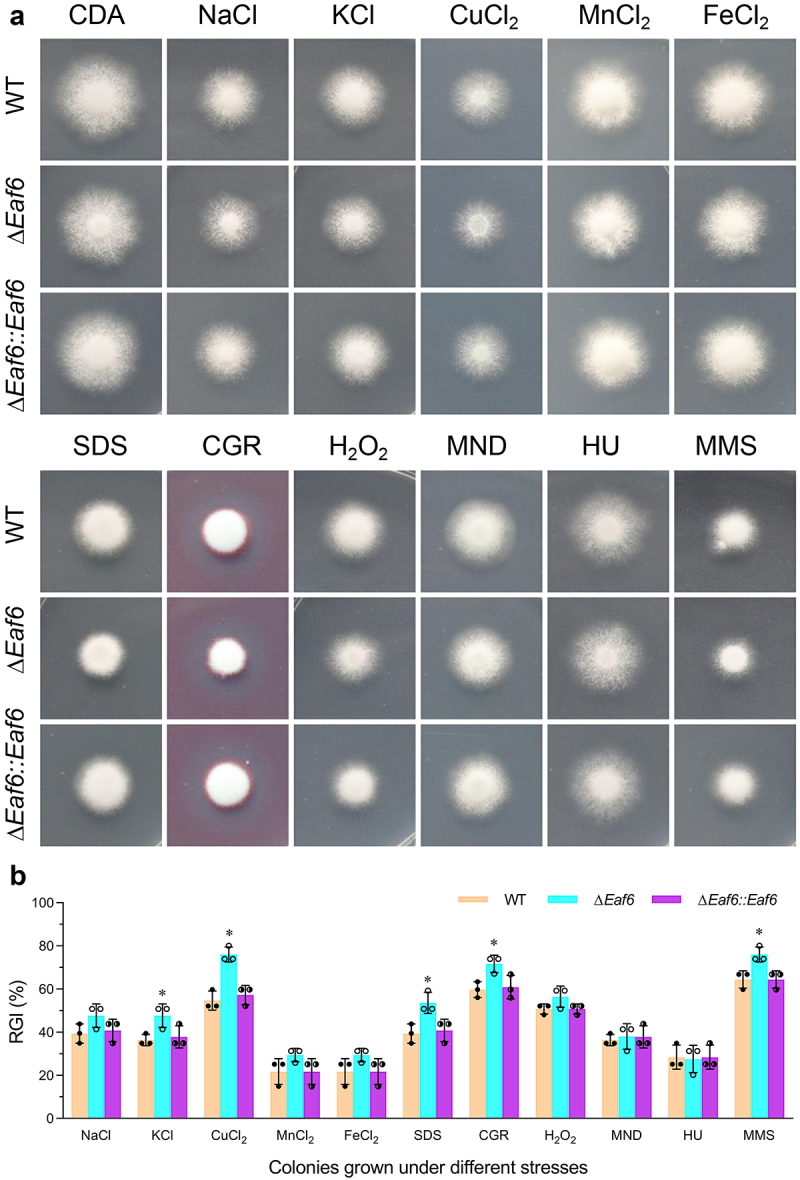
Impact of *BbEaf6* deletion on multi-stress tolerance in *B. bassiana*.

To investigate the role of BbEaf6 in multi-stress tolerances, wild type, Δ*BbEaf6*, and complemented strain (Δ*BbEaf6:BbEaf6*) were grown for 8 days in various media, including Czapek-Dox Agar (CDA), and CDA modified with different stressful agents. The dual images of fungal colonies and relative growth inhibition index (RGI) calculated based on the diameters were presented in Figure 2(a,b). As indicated in the results, compared to wild type, the deletion of *BbEaf6* resulted in reduced fungal tolerances to osmotic stresses, cell wall perturbing agents, and DNA damage stresses. For instance, although the Δ*BbEaf6* mutant showed no obvious difference in NaCl, MnCl_2_ and FeCl_2_, the loss of *BbEaf6* resulted in decreased tolerances to two different ionic stress agents (KCl and CuCl_2_) by ~11% and ~21%, respectively. Moreover, in the Δ*BbEaf6* strains, the fungal sensitivity of two different cell wall perturbing agents, SDS and Congo Red, were significantly increased by ~14% and ~12%. Additionally, the Δ*BbEaf6* mutant exhibited increased (~12%) sensitivity to the DNA damage-causing agent, methyl methanesulfonate (MMS), although unaffected by sensitivity to another DNA synthesize inhibitor (HU, hydroxyurea). However, the deletion of *BbEaf6* exerted on insignificant impact on the fungal tolerance to two different oxidant agents (H_2_O_2_ and menadione), as indicated in Figure 2(a,b).

(a, b) Representative images and relative growth inhibition (RGI) of fungal colonies grown at 25°C for 8 days on CDA media supplemented with various stress-inducing agents: NaCl (0.4 M), KCl (0.4 M), CuCl_2_ (2 mM), MnCl_2_ (4 mM), FeCl_2_ (4 mM), H_2_O_2_ (2 mM), menadione (MND; 0.02 mM), sodium dodecyl sulphate (SDS, 100 μg/ml), Congo red (CGR; 10 μg/ml), hydroxyurea (HU; 10 mM), and methyl methanesulfonate (MMS; 0.05%).

(Tukey’s honestly significant difference test, *p* < 0.05).

### Role of BbEaf6 in hyphal septation and cell cycle control

**Figure 3.**
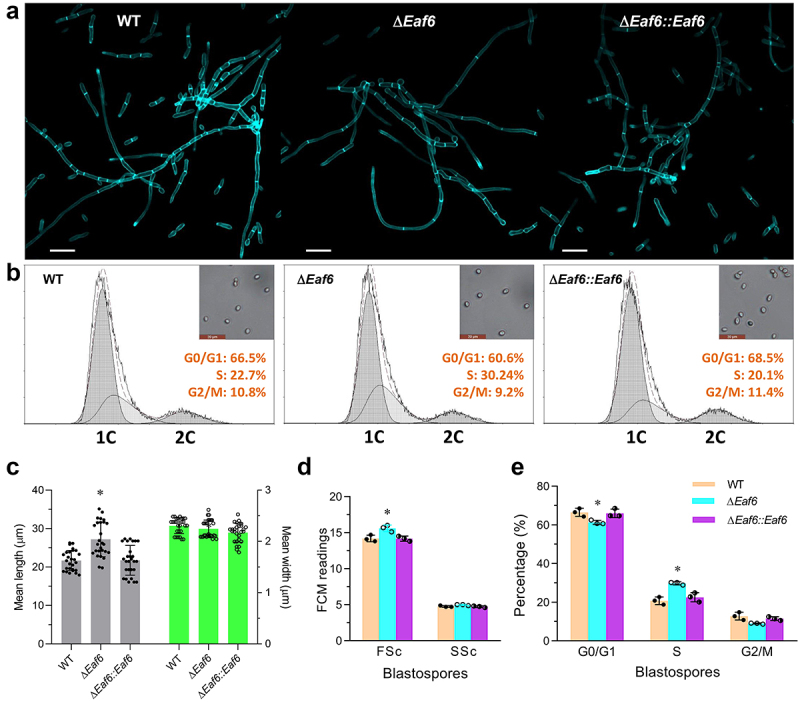
Contributions of *Eaf6* in hyphal septation and cell cycle control in *B. bassiana*.

To determine the role of BbEaf6 in submerged hyphal development, all strains including wild type, Δ*BbEaf6*, and complemented strain were inoculated in Sabouraud dextrose broth (SDB) for 72 hour. Hyphal cells were collected and stained with the cell wall-specific dye calcofluor white, followed by microscopic examination. As revealed in Figure 3(a), the fungal cells of Δ*BbEaf6* mutant strain showed obviously altered hyphal septation patterns compared to control strains. The average length of unicellular cells from Δ*BbEaf6* hyphae were significantly increased by ~26% than wild-type strain, while with no dramatic alteration in cell width (Figure 3(c)).

(a) Microscopic images (scale bars: 20 µm) showing hyphal cells collected from 72-hour-old SDB cultures and stained with calcofluor white (CFW). (b) Flow cytometry analysis for cell size (FSc) and density (SSc) of blastospores collected from 3-day-old nitrogen-limited broth (NLB) cultures. (c) Mean cell length and width of hyphal cells stained with calcofluor white from each strain. (d, e) Flow cytometry analysis for the distribution of cell cycle phases of blastospores collected from 3-day-old nitrogen-limited broth (NLB) cultures.

(Tukey’s honestly significant difference test, *p* < 0.05).

To examine the potential effects of BbEaf6 on cell cycle progression, the Δ*BbEaf6* mutant and control strains were incubated for 3 days at 25°C in nitrogen-limited broth (NLB) used to generate unicellular blastospores. The resulting cells were analysed by flow cytometry after staining with the DNA-specific dye propidium iodide. Compared to the wild type, blastospores derived from the Δ*BbEaf6* mutant strain showed a slight increase (~10%) in size but no change in cell density (Figure 3(d)). The Δ*BbEaf6*-derived cells exhibited significantly shorter (by 8%) G0/G1 phase (~60.6%), but longer (by 30%) S phase (~30.24%), while unaffected G2/M phase times (~9.2%) (Figure 3(b,e)), indicating a pronounced S phase. These results revealed the vital role of BbEaf6 in cell cycle control and hyphal septation.

### BbEaf6 is indispensable for fungal virulence

**Figure 4.**
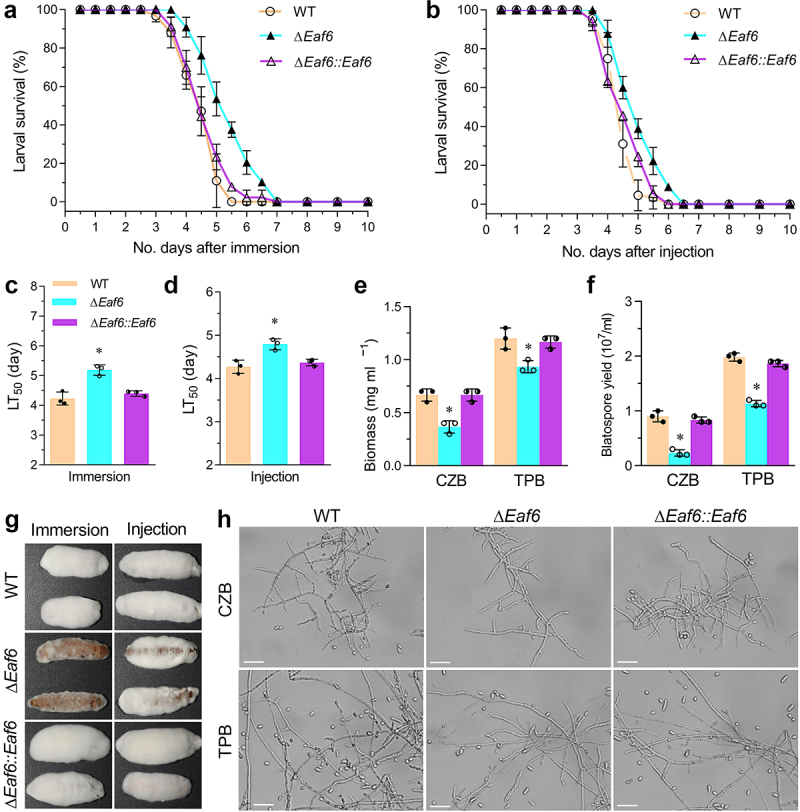
Impact of *Eaf6* deletion on fungal virulence upon insect host in *B. bassiana*.

To assess the role of BbEaf6 in *B. bassiana* virulence, insect bioassays using Greater wax moth, *Galleria mellonella*, larvae as hosts were conducted via both topical application bioassays (immersion) and intra-haemocoel injection bioassays (injection), with the median lethal time for killing 50% of the target hosts (LT_50_) calculated (Figure 4(a–d)). Overall, compared to wild type, the Δ*BbEaf6* strains exhibited decreased efficiency in killing insect host both in topical bioassays and in intra-haemocoel injection assays. In topical bioassays, the average of LT_50_ for the Δ*BbEaf6* strains was ~5.19 days, which was significantly prolonged by ~23% compared to the wild-type strain. While in intra-haemocoel injection assays, the average of LT_50_ for the Δ*BbEaf6* strains was ~4.79 days, which was prolonged by ~12%. Additionally, after 4 days post-death, the fungal outgrowths observed in the cadavers of theΔ*BbEaf6* strains were noticeably reduced compared to the control strains, indicating the attenuated ability to penetrate insect cuticle (Figure 4(g)).

(a–d) Survival trends and median lethal time (LT_50_) estimates of *Galleria mellonella* larvae after topical application (immersion) and intrahemocoel injection, respectively. (e, f) Biomass levels and blastospore yields are quantified respectively from the 3-day-old submerged cultures in Czapek-Dox broth (CZB) and trehalose-peptone broth (TPB). (g) Images depicting fungal outgrowths on the surfaces of cadavers 4 days post-death. (H) Microscopic images (scale: 20 μm) showing the submerged hyphae and blastospores from the 3-day-old cultures in CZB and TPB, respectively.

(Tukey’s honestly significant difference test, *p* < 0.05).

The attenuated intra-haemocoel virulence suggested that the transition of penetrating hyphae to unicellular blastospores was delayed or impeded in the Δ*BbEaf6* mutant. To test this hypothesis, we quantified the biomass and blastospore production after 3 days of growth in CZB and trehalose-peptone broth (TPB), a modified CZB that mimics insect haemolymph. Correspondingly, the Δ*BbEaf6* mutant showed significantly decreased total biomass in CZB (~45%) and TPB (~22%) media compared to the wild type and complemented mutants (Figure 4(e)). In terms of blastospore production, the Δ*BbEaf6* mutant was even more affected, showing decreased blastospores in CZB (~64%) and TPB (~43%) cultures (Figure 4(f)). Based on the biomass and blastospore yield, the dimorphic transition rate in the Δ*BbEaf6* mutant was decreased in CZB (~53%) and TPB (~26%) cultures compared to wild type. Under microscopy, the capacity of blastospores in the Δ*BbEaf6* mutant was obviously much less than in the control strains inoculated in the two different media above (Figure 4(h)). These results suggested that the deletion of *BbEaf6* affected the fungal growth in the insect body.

### Role of BbEaf6 in global gene transcription

**Figure 5.**
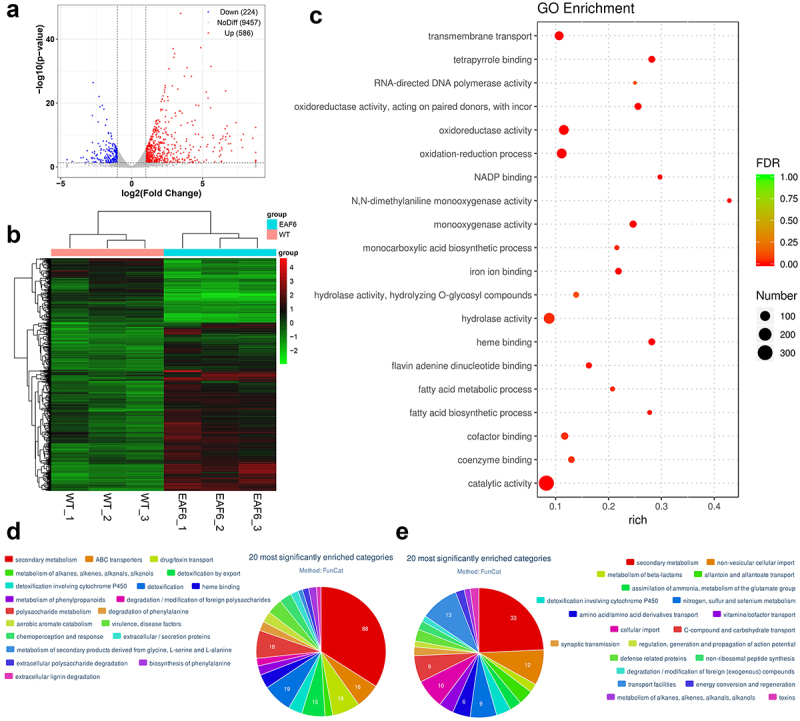
The global regulatory role of BbEaf6 in gene transcription *B. bassiana*.

To investigate the potential gene targets regulated by BbEaf6, comparative transcriptomic analyses were conducted to compare Δ*BbEaf6* and wild-type cells using triplicate biological samples, as detailed in the Materials and Methods section. A total of 810 differentially expressed genes (DEGs) were identified between the Δ*BbEaf6* mutant and wild type (Table S2). Among these DEGs, 224 DEGs were down-regulated [(Log_2_ (ratio): −4.55 to −1.00)], while 586 DEGs were up-regulated [(Log_2_ (ratio): +1.00 to +8.75)] in the Δ*BbEaf6* mutant compared to the wild type (Figure 5(a,b), Table S2).

(a) Distributions of *p* values and ratios for the genes are significantly up-regulated, down-regulated and not differentially regulated (No-Diff) in Δ*Eaf6* versus wild type. (b) Cluster analysis of differentially expressed genes (DEGs) in Δ*Eaf6* versus wild type. (c) Top 20 Gene Ontology (GO) terms are enriched based on the differentially expressed genes (DEGs) in Δ*Eaf6* versus WT. (d, e) Functional annotation for the top 20 functional categories of significantly up- or down-regulated genes in Δ*Eaf6* versus WT using the FunCat database.

Gene Ontology (GO) enrichment analyses were performed to identify gene functions that are significantly regulated in the Δ*BbEaf6* mutant relative to the wild type (Table S3). Among the top 20 enriched GO terms (Figure 5(c)), 314 DEGs were involved in catalytic activity, 125 DEGs were in hydrolase activity, 96 DEGs were in oxidoreductase activity and oxidation-reduction processes, and 71 DEGs were in transmembrane transport. Followed were those involved in cofactor binding (37), monooxygenase activity (35), haem binding (31), tetrapyrrole binding (31), iron ion binding (28), coenzyme binding (27), and flavin adenine dinucleotide binding (21). Moreover, the other DEGs were related to NADP binding (11), monocarboxylic acid biosynthetic process (11), fatty acid metabolic process (11), fatty acid biosynthetic process (10), N,N-dimethylaniline monooxygenase activity (9), and RNA-directed DNA polymerase activity (7), etc.

FunCat category classification was also performed to classify the up-regulated genes and down-regulated genes in the Δ*BbEaf6* mutant relative to the wild type (Table S4, S5). There were 178 up-regulated genes annotated in 37 different categories (Figure 5(d)). Among the 20 most significantly enriched categories, 88 DEGs were involved in secondary metabolism, followed by 19 DEGs in detoxification; 18 DEGs in drug/toxin transport; 18 DEGs in polysaccharide metabolism; 16 DEGs in ABC transporters; and 15 DEGs in detoxification by export. Moreover, the other DEGs were classified in detoxification involving cytochrome P450 (9), haem binding (9), chemoperception and response (8), aerobic aromate catabolism (7), and metabolism of phenylpropanoids (6), degradation of phenylalanine (6), etc. On the contrary, there were 71 down-regulated genes annotated in 21 different categories (Figure 5(e)). Among the 20 most significantly enriched categories, 33 DEGs were involved in secondary metabolism, followed by 13 DEGs in transport facilities; 12 DEGs in non-vesicular cellular import; and 10 DEGs in cellular import. Moreover, the other DEGs were classified in nitrogen, sulphur, and selenium metabolism (9), C-compound and carbohydrate transport (9), amino acid/amino acid derivatives transport (6), allantoin and allantoate transport (5), detoxification involving cytochrome P450 (5), and vitamin/cofactor transport (5), etc. These trancriptional changes indicating a global regulatory role of BbEaf6 in gene regulation in *B. bassiana*.

## Discussion

Protein acetylation has been regarded as a crucial regulatory mechanism in fungal infection process [33]. Histone acetyltransferases (HATs), which are responsible for lysine acetylation, have been proved to play vital roles in various pathogenic fungi, including different kinds of animal/human pathogens and plant pathogens [34–38]. In *B. bassiana*, five HATs, including members of the GNAT and MYST superfamily, have been studied regarding their roles in growth and virulence. Notably, Gcn5, Elp3, Spt10, Mst2, and Rtt109 have demonstrated varying contributions to cell morphogenesis and virulence [27–31]. However, the function of the NuA4 HAT complex, particularly its effects on filamentous fungi development and virulence, remains poorly understood. Attempts to delete the catalytic subunit Esa1 in *B. bassiana*, similar to yeast cells, were unsuccessful, suggesting its essentiality in filamentous fungi. Likewise, efforts to knock out the NuA4 subunit Elp1 also failed. However, successful knockout of Eaf6 in *B. bassiana* provided insight into its function in this fungus, shedding light on the role of the NuA4 HAT complex in fungal biology and pathogenicity.

The homologs of Eaf6 are integral components of the NuA4 and NuA3 complexes in yeast, as well as the MOZ/MORF complexes in humans, where they participate in transcriptional activation, gene regulation, and cell cycle control [4,6]. In this study, we identified a single Eaf6 homolog in *B. bassiana*, which, as anticipated, exhibited predominant nuclear localization, a characteristic shared with the plant pathogen *F. graminearum* [20]. Notably, the deletion of *BbEaf6* in *B. bassiana* resulted in differential regulation of a moderate number of genes (810), indicating its involvement in transcriptional regulation. Interestingly, the overall growth defect observed in the Δ*BbEaf6* mutant was less severe compared to that reported in yeast [15]. Specifically, we observed a higher number of upregulated genes (586) compared to downregulated genes (224) in the Δ*BbEaf6* mutant strain, suggesting a potential suppressor role for BbEaf6 in gene regulation.

Conidia serve as the primary active ingredient in insect biological control applications utilizing *B. bassiana*, and maintaining their viability is crucial for effective pest control [22]. In the Δ*BbEaf6* mutant, we observed delayed conidiation and reduced conidial yield, likely attributable to the significantly prolonged S-phase observed in Δ*BbEaf6* cells. Proper cell cycle regulation and hyphal septation are known to be essential for both vegetative growth and spore development [39]. Furthermore, RNA-Seq analysis of the Δ*BbEaf6* mutant revealed a wide range of differentially expressed genes (DEGs) involved in asexual development and cell cycle progression. For instance, the expression of two crucial factors for asexual development, AbaA and FlbC [40,41], was significantly regulated in the Δ*BbEaf6* mutant, likely contributing to the impaired sporulation observed. More specifically, the transcription of AbaA was downregulated due to the deletion of *BbEaf6*, while FlbC was upregulated. Additionally, the expression of several protein kinases implicated in DNA processes and cell cycle regulation were differentially regulated in the Δ*BbEaf6* mutant. These included members of the CAMK family, elongation factor-2 kinase EFK-1B, and ribose-phosphate pyrophosphokinase [42,43]. These findings highlight the intricate regulatory network orchestrated by BbEaf6 in governing fungal development and cellular processes.

Conidial tolerance to various stresses and complex environmental conditions is crucial for the viability of fungal conidia [23]. In the Δ*BbEaf6* mutant, we observed decreased conidial tolerance across different types of stressors, particularly ionic stress, cell wall-disturbing stress, and DNA damage stress. This reduced tolerance is correlated with significantly downregulated expression of numerous transporter genes in the Δ*BbEaf6* mutant, including 24 MFS transporters, 12 ABC transporters, four OPT oligopeptide transporters, and two ion transporters. However, the two genes associated with cell wall integrity and ion homoeostasis were significantly upregulated, including the cell wall stress-responsive component WSC4D and P-type IIC/NK Na+/K±ATPases (NK1) [44,45]. Furthermore, several genes involved in DNA replication and repair were significantly downregulated in the Δ*BbEaf6* mutant, including DNA repair protein Rad57 [46], elongation factor-2 kinase, and RNase H domain containing protein. These transcriptional alterations likely contribute to the impaired conidial tolerance to various stressful conditions observed in the Δ*BbEaf6* mutant. A comprehensive understanding of these molecular mechanisms sheds light on the role of BbEaf6 in orchestrating fungal responses to environmental stressors, thereby influencing conidial viability and fungal fitness.

Our findings also underscore the essential role of BbEaf6 in the full virulence of *B. bassiana*, encompassing both the ability to breach the insect cuticle and subsequent growth and evasion of the host immune response within the haemocoel. The deletion of *BbEaf6* resulted in significantly differential regulation of numerous genes crucial for cuticle penetration and haemocoel infection processes in *B. bassiana*. For instance, some protein/peptide degrading enzymes were found to be downregulated, including four peptidases, one aspartic protease, a subtilase-like protein and lipase 2 precursor. Also, the expression of two subtilisin-like proteases (Pr1A and Pr1C), which are pivotal for cuticle degradation and invasion [47], were differentially regulated in the Δ*BbEaf6* mutant. Additionally, the expression of hydrophobins, essential for fungal conidial hydrophobicity and adhesion to the host cuticle [48], and LysM domain proteins, known to suppress chitin-induced immunity and contribute to fungal virulence in *B. bassiana* [49], was also found to be differentially regulated in the Δ*BbEaf6* mutant.

In summary, our study elucidates the functional aspects of BbEaf6, highlighting its role in multi-stress sensitivity, delayed cell cycle progression and conidiation, and impaired virulence as major consequences of BbEaf6 loss. These insights provide valuable knowledge for understanding the molecular mechanisms underlying fungal pathogenesis and contribute to the development of novel strategies for insect pest management.

## Supplementary Material

Supporting information20240429.xlsx

Eaf6 Supporting Information20240428.docx

## Data availibility statement

All transcriptomics data have been deposited in the Sequence Read Archive (SRA) on NCBI (https://www.ncbi.nlm.nih.gov/) and are openly available with the dataset identifiers PRJNA721731.
