# Supplementary material for: Functional characterization of BbEaf6 in Beauveria bassiana: Implications for fungal virulence and stress response
Source: Virulence. 2024 Jul 31;15(1):2387172. doi: 10.1080/21505594.2024.2387172 (PMC11299629; doi:10.1080/21505594.2024.2387172)
Supplement: Eaf6 Supporting Information20240428.docx [file KVIR_A_2387172_SM3973.docx]

**Supporting Information**

**Functional characterization of BbEaf6, a homolog of Eaf6 in *Beauveria bassiana*: Implications for fungal virulence and stress response**

**Qing Cai^1*^, Juan**-**Juan Wang^2^, Jia-Tao Xie^1^,** **and Dao-Hong Jiang^1^**

^1^ State Key Laboratory of Agricultural Microbiology, College of Plant Science and Technology, Huazhong Agricultural University, Wuhan, Hubei, 430070, China

^2^ School of Biological Science and Biotechnology, University of Jinan, Jinan, Shandong, 250022, China

.

*** Corresponding author:** Qing Cai, E-mail: caiqing@mail.hzau.edu.cn.


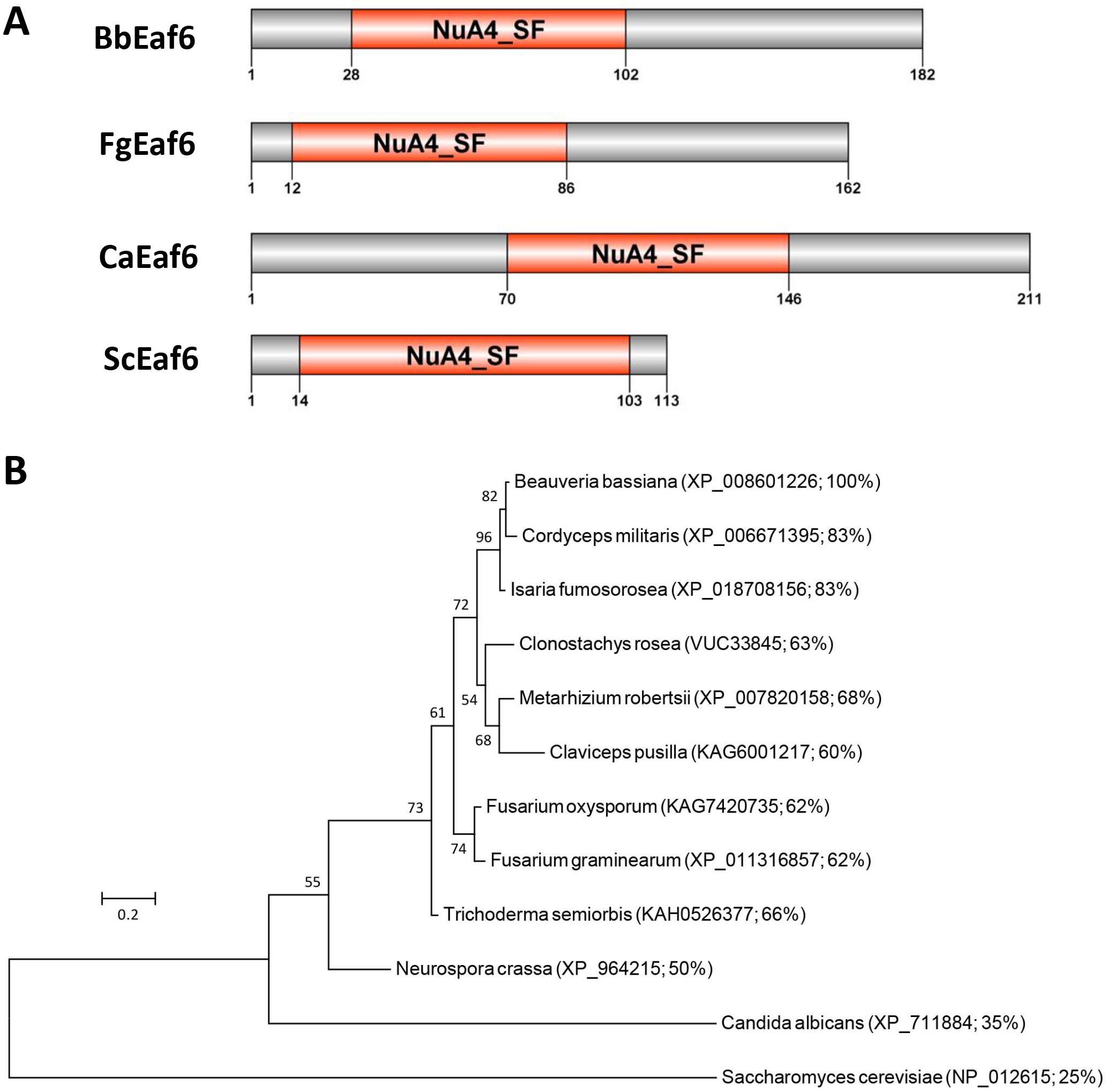


**Fig. S1.** Conserved domain and phylogenetic relationship of *B. bassiana* Eaf6 with the homologs found in other representative organisms. (**A**) Sequence features of Eaf6 homologs found in *Beauveria bassiana* (Bb), *Candida albicans* (Ca), *Fusarium graminearum* (Fg), and *Saccharomyces cerevisiae* (Sc). The domain of each protein was predicted at <https://blast.ncbi.nlm.nih.gov/Blast.cgi>. (**B**) Phylogenetic relationship of *B. bassiana* Eaf6 with the homologs found in other representative fungi. A neighbor-joining method in MEGA7 at <http://www.megasoftware.net> was used in the phylogenetic analysis. Each fungal name is followed by the NCBI accession code of each protein and its sequence identity (%) to *B. bassiana* Eaf6 in parentheses. Jones-Taylor-Thornton (JTT) model was used with 1000 bootstrap replications in uniform rates. Scale bar: branch length proportional to genetic distance assessed with the neighbor-joining method.

**
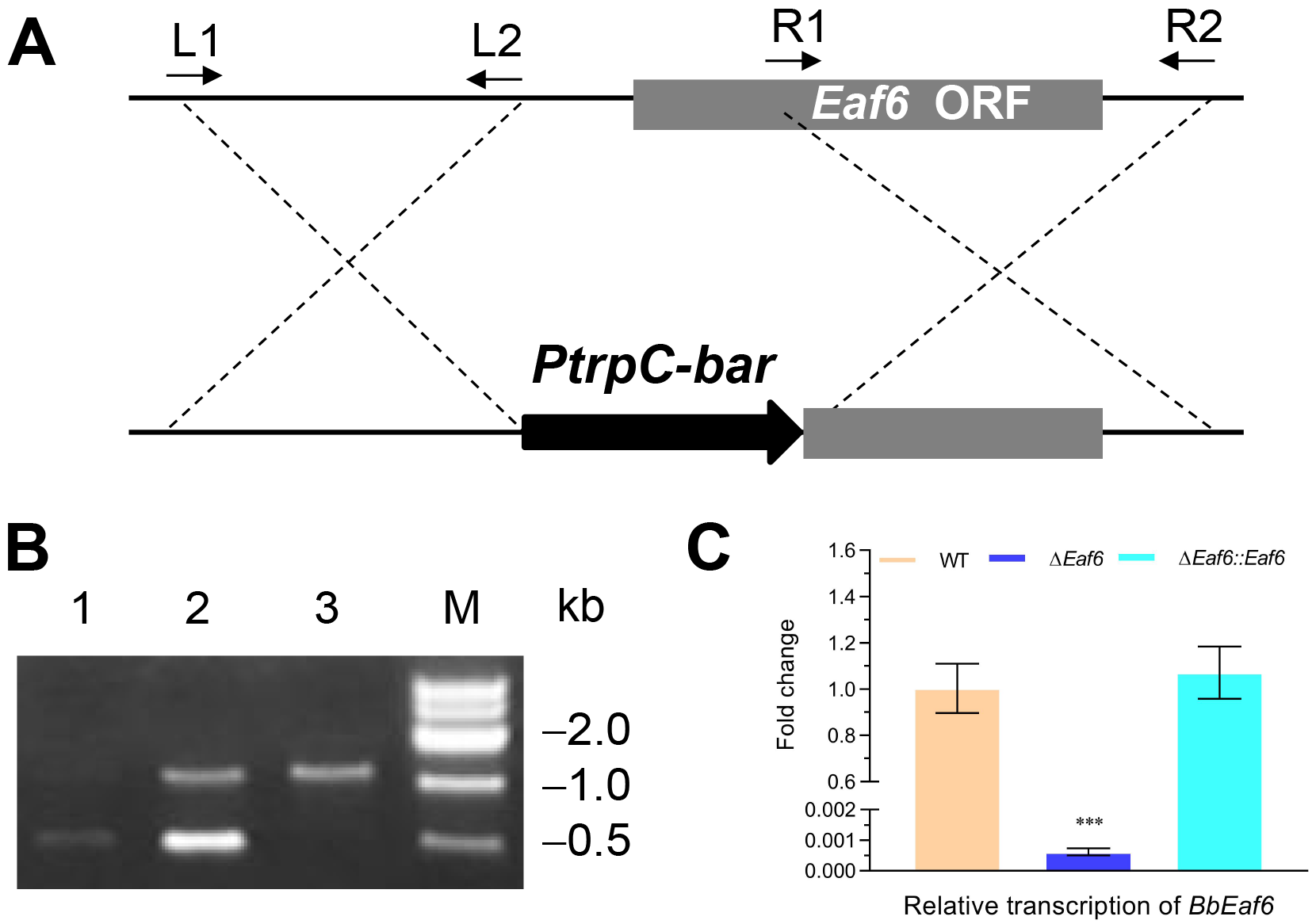
**

**Fig. S2** Generation and identification of *B. bassiana Eaf6* mutants. (**A**) Schematic diagram for the strategy of *Eaf6* deletion. (**B & C**) The *Eaf6* mutants were identified via PCR (B) and real-time PCR (C) analyses with paired primers and amplified probe (Table S1). Lanes 1: wild-type; Lanes 2: *ΔEaf6::Eaf6* mutant; Lanes 3: *ΔEaf6* mutant*.*

**Table S1.** Paired primers designed for manipulation of *Eaf6* and identification of its mutants in *B. bassiana*.

| Primers | Paired sequences (5'-3')* | Purpose |
| --- | --- | --- |
| Eaf6-LC-F/R | CCCGGGACTAGTGATATCATGGCAGACAACAAGCGC / CTTGCTCACCATGAATTCCTTTCTCGAAGCGCCGAAC | Cloning *Eaf6* cDNA(546 bp) |
| Eaf6-UP-F/R | AAAAAGAATTCAACATTCAGATTCAGCTTACCG / AAAAACCCGGGATGAAGAGGAGGTTAGAGTGGC | Cloning *Eaf6* 5′-end (1509 bp) |
| Eaf6-DN-F/R | AAAAACTCGAGCTGCTTACCTAGAGAATACGCC / AAAAAACTAGTCATTCCCCTCATCTACACCTTA | Cloning *Eaf6* 3′-end (1442 bp) |
| Eaf6-Com-F/R | ggggACCACTTTGTACAAGAAAGCTGGGTNATGGCAGACAACAAGCGC / ggggACCACTTTGTACAAGAAAGCTGGGTNGCAGCATACGCATCTCAT | Cloning full-length *Eaf6* (3528 bp) |
| Eaf6-ID-F/R | GCTGTGCTGTTAGTCGTCTGTG / AGTTGTCGAAACCGGTGATG | PCR detecting *Eaf6* |
| Eaf6-qRT-F/R | CGGGAACTGGTTGAGAAACG / TGGGCCGGTAGGAAATGG | q-PCR detecting *Eaf6* (229 bp) |

* Underlined regions denote the restriction enzyme sites used for cloning *Eaf6* cDNA (*Spe*I/*EcoR*I) or deleting *Eaf6* (*EcoR*I/*Xma*I and *Xho*I/*Spe*I) and the fragments of gateway exchange for *Eaf6* complementation.
